# Sex differences of sequential changes in coronary blood flow and microvascular function in patients with suspected angina

**DOI:** 10.1007/s00392-023-02358-2

**Published:** 2023-12-19

**Authors:** So Ree Kim, Mi-Na Kim, Dong-Hyuk Cho, Hee-Dong Kim, Sung A. Bae, Hack-Lyoung Kim, Myung-A Kim, Kyung-Soon Hong, Wan Joo Shim, Seong-Mi Park

**Affiliations:** 1 Division of Cardiology, Korea University Anam Hospital, 73 Goryeodae-ro Seongbuk-gu, Seoul, 02841 Republic of Korea; 2 Division of Cardiology, Soonchunhyang University Hospital, 31, Suncheonhyang 6-gil, Dongnam-gu, Cheonan-si, Chungcheongnam-do Republic of Korea; 3 Division of Cardiology, Yongin Severance Hospital, 363, Dongbaekjukjeon-daero, Giheung-gu, Yongin-si, Gyeonggi-do Republic of Korea; 4 Cardiovascular Center, Seoul National University Boramae Hospital, 20, Boramae-ro 5-gil, Dongjak-gu, Seoul, Republic of Korea; 5 Division of Cardiology, Hallym University Chuncheon Sacred Heart Hospital, 77, Sakju-ro, Chuncheon-si, Gangwon-do Republic of Korea

**Keywords:** Angina, Coronary blood flow, Coronary microvascular function, Coronary flow velocity reserve, Sex differences

## Abstract

**Aims:**

This study evaluated the sex differences of sequential changes in coronary blood flows and microvascular function in patients with suspected angina but with no obstructed coronary arteries.

**Methods:**

A total of 202 consecutive patients who experienced chest pain but had no significant coronary artery stenosis and who underwent adenosine stress echocardiography were included in the study. Coronary blood flow (CBF) velocities were measured at 1, 2, and 3 min after adenosine infusion.

**Results:**

The mean age was 61 years, and 138 (68%) were women. Approximately 40% of patients had coronary microvascular dysfunction (CMD, coronary flow velocity reserve < 2.3), with women exhibiting higher CMD prevalence. The left ventricular (LV) mass index was similar between men and women, while women exhibited higher baseline rate pressure products (RPP). At baseline, coronary blood flow velocities were similar between the sexes. However, CBF velocities in women gradually increased during the examination; and in men, the increase was abrupt and steep during the early stages of examination (p = 0.015 for interaction between time and sex), even with similar RPP in stress. Coronary flow velocity reserve was steadily lower in women compared to men (1 min, 2.09 ± 0.86 vs 2.44 ± 0.87; 2 min, 2.39 ± 0.72 vs 2.63 ± 0.85; 3 min, 2.45 ± 0.70 vs 2.68 ± 0.73).

**Conclusions:**

In patients with suspected angina but with no obstructed coronary arteries, CMD was especially prevalent among women. Women exhibited higher oxygen consumption, while exhibiting slower and gradual increases in CBF velocities. Conversely, men exhibited faster and steeper increases in CBF velocities even with similar RPP in stress.

**Graphical abstract:**

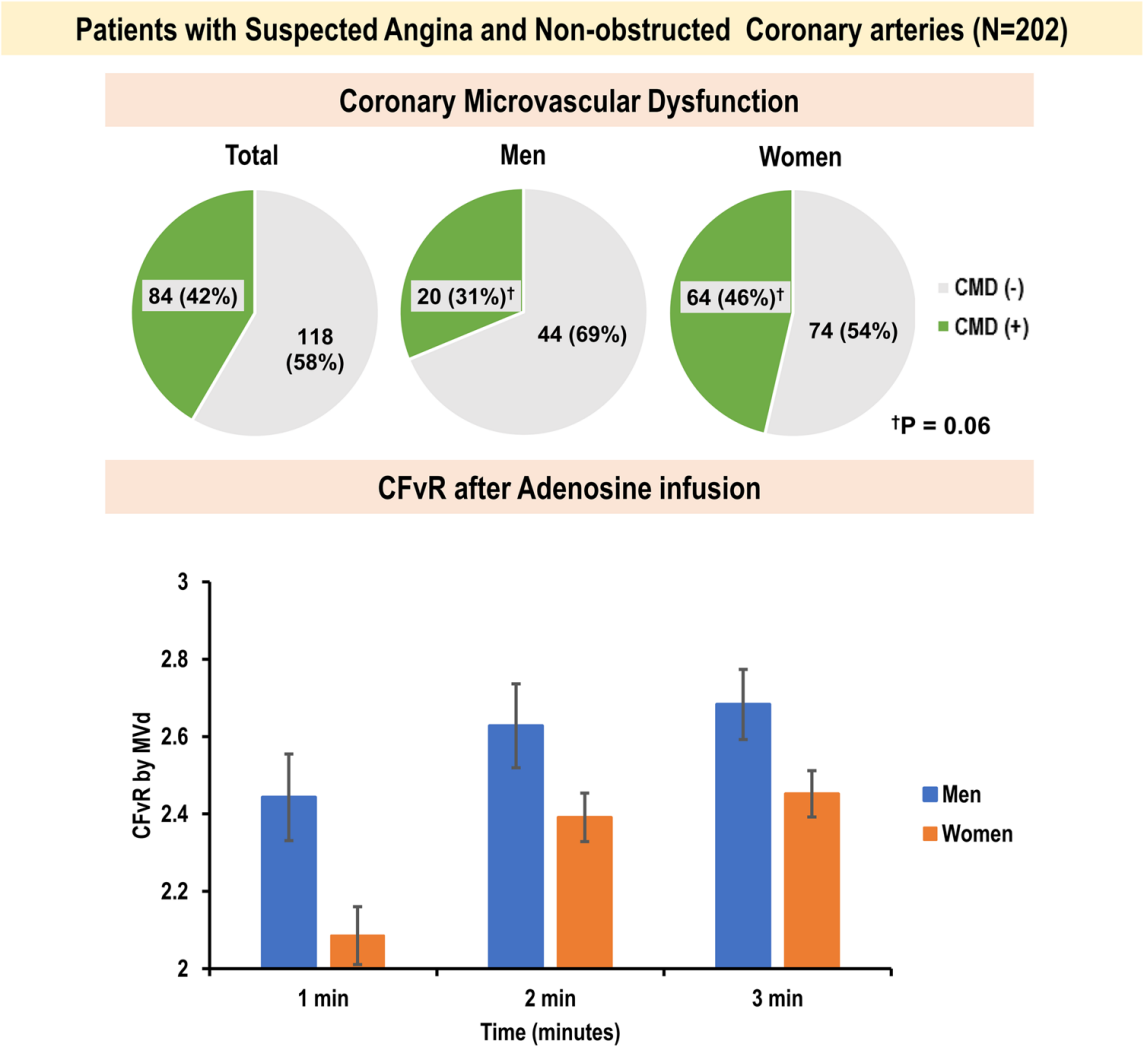

**Supplementary Information:**

The online version contains supplementary material available at 10.1007/s00392-023-02358-2.

## Background

A considerable number of patients with symptoms or signs of ischemic heart disease without obstructed coronary arteries (Ischemia and No Obstructive Coronary Artery Disease, INOCA) have coronary microvascular dysfunction (CMD) [1–3]. CMD is a major endotype of INOCA and can be caused by functional and structural changes to the coronary microvasculature as well as abnormal vasodilation due to endothelial dysfunction or vasospasm induced by various stimuli [4]. Recent studies have suggested that CMD is a major component in the pathophysiology of heart failure with preserved ejection fraction [5–7]. CMD is also associated with poor exercise capacity and adverse outcomes, including death, nonfatal myocardial infarction, nonfatal stroke, and hospitalization for heart failure [8, 9].

While microvascular dysfunction can occur in men and women, research has shown that women are more susceptible to CMD [10]. Women with CMD may experience different and a more diverse set of symptoms than men, which can hinder diagnosis and treatment [11]. Furthermore, women with CMD may have a worse prognosis than men, with higher rates of adverse cardiovascular events such as heart attack, stroke, heart failure, and death [6, 12]. Women have relatively smaller cardiac chambers resulting in lower stroke volumes; therefore, they have a higher left ventricular ejection fraction (LVEF) and resting heart rate to maintain cardiac output [13]. In addition to structural differences, women may have increased resting coronary blood flow (CBF) [10]. However, the mechanism responsible for the sex difference in symptoms and poor outcomes of CMD remains unclear. In the present study, we investigate the sex differences of sequential changes in coronary blood flows and microvascular function in patients with suspected angina but with no obstructed coronary arteries.

## Methods

### Study population

From February 2018 to April 2021, 202 consecutive patients who experienced chest pain but with no obstructed coronary arteries were prospectively enrolled and underwent an adenosine stress echocardiography at Korea University Anam Hospital [Seoul, Korea]). The study protocol was reviewed and approved by the Institutional Review Board of the same institute (IRB number: 2017AN0358). Informed consent forms were signed by all enrolled patients. (ClinicalTrials.gov Identifier: NCT06076551).

Nonobstructive coronary artery stenosis was defined as coronary artery stenosis < 50% on coronary angiography or computed tomography. All patients showed insignificant coronary artery stenosis (% diameter stenosis, [0–30%]). Patients with sinus node dysfunction or symptomatic bradycardia, congenital heart disease, ≥ moderate valvular heart disease, active cancer, chronic renal failure (estimated glomerular filtration rate < 30 mL/min/1.73 m^2^), and chronic obstructive pulmonary disease were excluded.

### Coronary flow velocity reserve

**Fig. 1:**
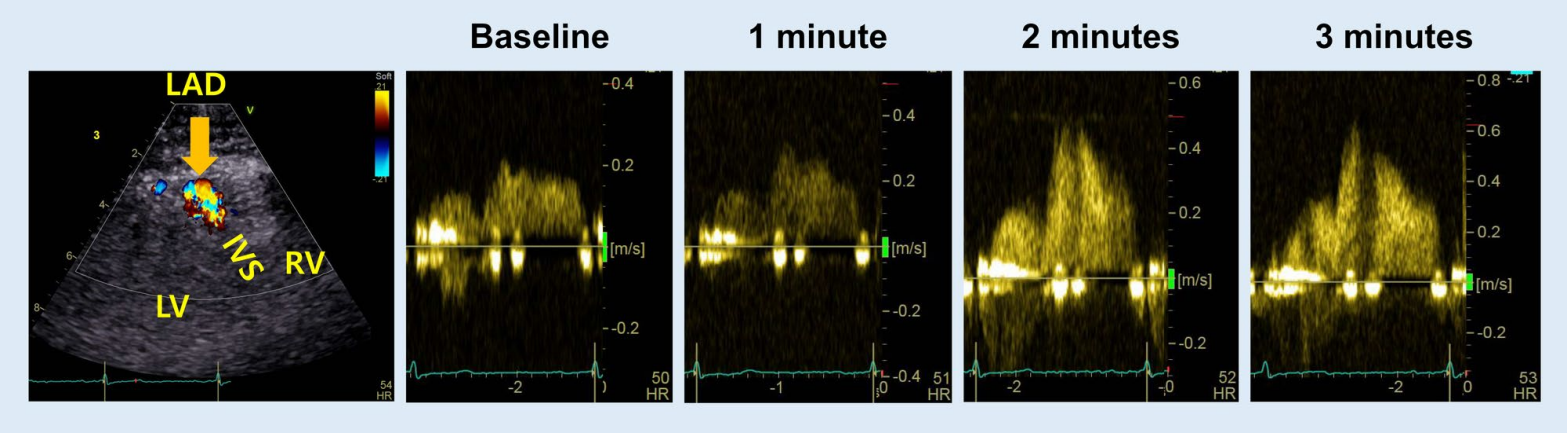
Transthoracic echocardiographic assessments of CBF velocities. The color Doppler flow of the distal left anterior descending artery was examined from the modified apical four-chamber view of the anterior interventricular groove. Pulsed-wave Doppler images were acquired at baseline, and 1, 2, and 3 min after adenosine infusion in the same part of the artery. *CBF* coronary blood flow, *IVS* interventricular septum, *LAD* left anterior descending artery, *LV* left ventricle, *RV* right ventricle

Transthoracic echocardiographic assessments were performed using an ultrasound device (Vivid E95, GE Healthcare, Liestal, Switzerland). Color Doppler flow of the distal left anterior descending artery was examined from the modified apical four-chamber view of the anterior interventricular groove [14]. Pulsed-wave Doppler registered blood flow velocity patterns using a sample volume (2–3.0 mm) placed on the color signal. The ultrasound beam was aligned parallel to the vessel flow. The velocity scale of color Doppler was set to 0.21 m/s. Coronary flow Doppler images were acquired at baseline and at 1, 2, and 3 min after adenosine infusion in the same part of the artery (Fig. 1) [15]. Anti-anginal medications, including calcium channel blockers, were discontinued before the study. Since 43 patients (21.3% of study population) were taking beta-blockers, these medications were discontinued for two days before the adenosine stress echocardiography. The peak and mean diastolic coronary flow velocities, diastolic deceleration time of coronary flow, and peak and mean systolic coronary flow velocities were measured. Coronary flow velocity reserve (CFvR) was defined as the ratio of peak to baseline mean diastolic velocity (MVd) of coronary flow. CMD was defined as impaired vasodilation of arterioles and a CFvR < 2.3 [3]. CFvR was independently evaluated by two cardiologists (SRK and MNK): intra-observer variability was 98.1% (95% confidence interval [CI] 92.8–99.5), and inter-observer variability was 95.1% (95% CI 80.9–98.8). The evaluation of this study was conducted using the average of the values measured by two cardiologists.

Arterial pressure and heart rate were measured at baseline and peak stress. Rate pressure product (RPP) is an indirect index of myocardial oxygen consumption and was defined as the product of systolic blood pressure and heart rate [16]. Comprehensive echocardiographic assessment, including left ventricular (LV) strain analysis, was performed at baseline and peak stress.

### Exercise capacity

The maximal tolerable treadmill exercise test was performed using the Bruce protocol to evaluate myocardial ischemia and exercise capacity. The treadmill exercise test was performed within one month of the adenosine stress echocardiography. Total exercise time in seconds, amount of work (according to the metabolic equivalent of task), and Duke treadmill score were assessed. ST segment deviation during exercise was defined as horizontal or down-sloping depression or elevation in leads without pathological Q waves, excluding aVR lead. The Duke treadmill score was calculated using the following equation [17]: Duke treadmill score = maximum exercise time (in minutes) − 5 × ST segment deviation in mm − 4 × angina index (where 0 = no angina, 1 = non-limiting angina, 2 = exercise limiting angina). A Duke score ≥ 5 indicates a low risk for cardiovascular events (predicted 4-year survival was 99%), while a score < − 10 indicates a high risk (predicted 4-year survival was 79%). A score between 4 and − 10 represents intermediate risk [17].

### Primary endpoint

The primary endpoint was defined as a composite outcome including all-cause mortality, nonfatal myocardial infarction, nonfatal stroke, and hospitalization for heart failure at the 1-year follow-up. During the follow-up period, 29 (14%) patients were lost to follow-up, with the majority being referred to other hospitals due to their stable medical condition.

### Statistical analysis

Categorical data were assessed using the chi-squared test or Fisher’s exact test, as appropriate. Continuous variables were compared using the Student’s *t*-test or the Mann–Whitney test where applicable. A linear mixed model was conducted to examine the interaction between sex and the temporal changes in MVd of coronary flow. For assessing sex differences at each time point, Bonferroni’s method was applied with corrected p-values. To identify independent predictors of CMD, we performed multivariable binary logistic regression analysis. The inclusion of covariates in the multivariable models was based on their significance in univariable analysis or clinical relevance to CMD. The covariates included in the multivariable analysis encompassed age, sex, body mass index, hypertension, diabetes, dyslipidemia, smoking status, baseline LV mass index, E/e′, global longitudinal strain (GLS), and RPP. To calculate the correlation between CFvR and exercise capacity, we utilized correlation analysis and presented the correlation coefficient (r). All probability values were two-sided, and significance was defined as p < 0.05. The statistical analyses were performed using SPSS version 20.0 (IBM Corporation, Armonk, NY, USA) and R version 4.0.3 (R Foundation for Statistical Computing, Vienna, Austria).

## Results

### Baseline clinical and laboratory characteristics

Of the 202 patients, 138 (68%) were women; the mean age was 61. Approximately one-half of the patients had hypertension (48%) and dyslipidemia (47%), and 12% had diabetes. Approximately one-fifth of the patients were smokers, 78.4% of them were men. Women exhibited higher levels of N-terminal pro-B-type natriuretic peptide (NT-proBNP) (58.1 [interquartile range (IQR) 27.7–98.6] vs. 22.5 [IQR 12.4–47.0) pg/mL; p = 0.001) despite higher usage of diuretics (19.5% vs. 12.5%; p = 0.305). Women also exhibited lower hemoglobin and hematocrit levels than men. Other baseline clinical and laboratory characteristics are summarized in Table 1.

**Table 1:** Baseline demographics and laboratory tests

	Total patients (N = 202)	Men (N = 64)	Women (N = 138)	*p* value
Age (years)	61.3 ± 9.7	60.3 ± 10.0	61.7 ± 9.5	0.352
Body surface area (m^2^)	1.68 ± 0.17	1.83 ± 0.13	1.61 ± 0.13	< 0.001
Body mass index (kg/m^2^)	25.0 ± 3.2	25.2 ± 2.5	24.9 ± 3.4	0.469
Hypertension, n (%)	97 (48.3)	30 (46.9)	67 (48.9)	0.907
Diabetes, n (%)	24 (11.9)	8 (12.5)	16 (11.7)	1.000
Dyslipidemia, n (%)	95 (47.3)	32 (50.0)	63 (46.0)	0.868
Current or ex-smoker, n (%)	37 (18.4)	29 (45.3)	8 (5.8)	< 0.001
Medication, n (%)				
Aspirin	53 (27.2)	24 (38.1)	29 (22.0)	0.028
Clopidogrel	15 (7.7)	8 (12.7)	7 (5.3)	0.123
Angiotensin inhibitor	67 (34.1)	21 (31.9)	46 (34.6)	0.752
Beta blocker	40 (20.3)	12 (19.0)	28 (20.9)	0.912
Calcium channel blocker	82 (41.8)	28 (44.4)	54 (40.6)	0.723
Diuretics	34 (17.3)	8 (12.5)	26 (19.5)	0.305
Oral hypoglycemic agents	22 (11.0)	8 (12.5)	14 (10.3)	0.824
Anti-lipid agents	105 (52.0)	36 (56.2)	69 (50.0)	0.499
Laboratory tests				
Hemoglobin (g/dL)	13.6 ± 1.3	14.6 ± 1.1	13.1 ± 1.0	< 0.001
Hematocrit (%)	40.2 ± 3.3	43.0 ± 2.9	38.9 ± 2.6	< 0.001
BUN (mg/dL)	15.3 (12.9–18.1)	15.6 (12.9–18.1)	15.1 (12.9–18.1)	0.842
eGFR (mL/min/1.73 m^2^)	84.8 (74.7–98.3)	84.6 (75.2–99.1)	84.8 (74.5–96.9)	0.731
hsCRP (mg/L)	0.64 (0.34–1.32)	0.68 (0.36–1.31)	0.61 (0.31–1.32)	0.507
Fasting glucose (mg/dL)	108.0 (100.0–121.0)	111.0 (101.0–125.0)	105.5 (99.0–120.0)	0.138
HbA1c (% of total Hb)	5.8 (5.5–6.3)	5.8 (5.5–6.2)	5.8 (5.5–6.3)	0.580
Uric acid (mg/dL)	4.8 (4.0–5.5)	5.3 (4.5–5.7)	4.3 (3.7–5.1)	0.008
Total cholesterol (mg/dL)	172.5 (149.0–207.0)	169.0 (144.0–186.0)	179.0 (152.0–217.0)	0.025
Triglyceride (mg/dL)	115.0 (83.5–158.0)	146.0 (97.5–201.5)	104.5 (81.0–143.5)	0.003
HDL-cholesterol (mg/dL)	50.0 (46.0–56.0)	46.0 (41.0–50.0)	53.5 (49.0–60.0)	< 0.001
LDL-cholesterol (mg/dL)	103.0 (74.5–127.5)	97.0 (71.5–119.5)	105.0 (77.5–135.0)	0.203

*BUN* blood urea nitrogen, *eGFR* estimated glomerular filtration rate, *HbA1c* glycated hemoglobin, *HDL* high density lipoprotein, *hsCRP* high-sensitivity C-reactive protein, *LDL* low density lipoprotein

### Echocardiographic characteristics

Data regarding comprehensive echocardiographic measurements at baseline and peak stress with adenosine are summarized in Table 2. At baseline, women exhibited smaller LV sizes than men. The mean LV mass index was similar (81.9 ± 15.2 vs. 83.8 ± 15.9 g/m^2^, p = 0.424), while the mean LVEF was higher in women (63.3 ± 5.7 vs. 60.7 ± 5.4%, p = 0.003). Higher early diastolic mitral inflow velocity with no difference in medial e′ velocity resulted in higher baseline E/e′ in women (9.7 [IQR 8.0–11.7] vs. 9.0 [IQR 7.1–11.0], p = 0.036). The LV GLS was higher in women (− 20.1 ± 2.4 vs. − 18.3 ± 2.4, p < 0.001).

**Table 2:** Echocardiographic parameters

	Total patients (N = 202)	Men (N = 64)	Women (N = 138)	*P*-value
Baseline				
LV mass index (g/m^2^)	82.5 ± 15.4	83.8 ± 15.9	81.9 ± 15.2	0.424
Relative wall thickness	0.39 (0.36–0.42)	0.38 (0.35–0.40)	0.39 (0.36–0.42)	0.250
LV end-diastolic volume index (mL/m^2^)	48.5 (42.3–55.3)	50.3 (43.9–58.3)	47.2 (40.6–52.8)	0.018
LV ejection fraction (%)	62.5 ± 5.7	60.7 ± 5.4	63.3 ± 5.7	0.003
Left atrial volume index (mL/m^2^)	26.7 (21.6–34.0)	25.4 (22.0–32.4)	27.1 (21.5–36.0)	0.400
Mitral E velocity (cm/s)	58.7 (48.0–73.7)	50.3 (44.2–63.3)	61.1 (51.1–76.1)	< 0.001
Deceleration time of mitral E velocity (ms)	208.0 (176.0–238.0)	216.5 (170.8–244.5)	203.0 (176.5–236.6)	0.311
Mitral A velocity (cm/s)	65.9 (56.0–74.0)	60.7 (52.0–71.5)	67.0 (58.0–76.0)	0.012
E/A	0.87 (0.72–1.14)	0.84 (0.71–1.10)	0.89 (0.73–1.16)	0.339
Medial e′ velocity (cm/s)	6.4 ± 1.7	6.2 ± 1.6	6.5 ± 1.8	0.321
Medial E/e′	9.3 (7.8–11.3)	9.0 (7.1–11.0)	9.7 (8.0–11.7)	0.036
Right ventricular systolic pressure (mmHg)	29.2 (26.7–32.5)	29.0 (26.4–32.0)	29.5 (27.0–33.0)	0.666
Global longitudinal strain (%)	− 19.5 ± 2.5	− 18.3 ± 2.4	− 20.1 ± 2.4	< 0.001
Peak echocardiography				
LV end-diastolic volume index (mL/m^2^)	48.2 (42.5–53.7)	50.6 (44.1–56.5)	47.2 (40.8–52.8)	0.026
Cardiac output (L/min)	4.44 (3.87–5.16)	4.68 (4.08–5.35)	4.33 (3.77–4.96)	0.024
LV contractile reserve by Simpson (%)	9.1 (− 0.1–21.9)	7.9 (− 2.8–21.0)	10.2 (0.2–22.4)	0.329
LV ejection fraction (%)	69.7 (64.9–72.8)	65.4 (62.9–70.6)	70.9 (66.4–73.9)	< 0.001
Left atrial volume index (mL/m^2^)	29.6 (24.0–36.2)	30.0 (22.5–35.3)	29.4 (24.4–37.2)	0.479
Mitral E velocity (cm/s)	80.4 (70.1–93.0)	77.0 (65.6–85.7)	83.4 (71.1–95.8)	0.014
Deceleration time of mitral E velocity (ms)	201.0 (172.0–232.0)	199.0 (167.5–218.0)	203.0 (173.0–234.0)	0.531
Mitral A velocity (cm/s)	85.1 ± 20.0	76.7 ± 19.4	89.3 ± 18.9	< 0.001
E/A	0.92 (0.79–1.13)	0.97 (0.82–1.19)	0.88 (0.78–1.10)	0.077
Medial e′ velocity (cm/s)	7.3 (6.0–8.6)	7.2 (6.1–8.3)	7.3 (5.8–8.8)	0.723
Diastolic reserve (cm/s)	0.70 [− 0.10; 1.80]	0.74 [− 0.10; 1.80]	0.67 [− 0.16; 1.80]	0.676
Medial E/e′	11.0 (9.1–13.2)	10.5 (8.9–12.0)	11.1 (9.3–13.8)	0.102
Global longitudinal strain (%)	− 21.9 ± 2.5	− 20.7 ± 2.2	− 22.5 ± 2.5	< 0.001
% change of global longitudinal strain (%)	11.5 (6.5–18.2)	13.3 (7.3–18.2)	11.4 (6.2–17.8)	0.285

Diastolic reserve was defined as peak e′ − baseline e′

*A* late diastolic mitral inflow velocity, *E* early diastolic mitral inflow velocity, *e*′ early diastolic mitral annulus velocity, *LV* left ventricular

At peak stress with adenosine, LVEF and GLS were also higher in women, but no difference was seen in LV contractile reserve (10.2% [IQR 0.2–22.4] vs. 7.9% [IQR − 2.8–21.0], p = 0.329). No difference in percent GLS change was observed between the sexes. Although not statistically significant, the E/e′ of women at peak stress was numerically higher than that of men (11.1 [IQR 9.3–13.8] vs. 10.5 [IQR 8.9–12.0], p = 0.102). Diastolic reserve, which was defined as peak e′ − baseline e′, was similar in both sexes (0.67 m/s [IQR − 0.16–1.80] vs 0.74 m/s [IQR − 0.10–1.80], p = 0.676).

### Coronary blood flow

CBF parameters and serial changes over time after adenosine administration are summarized in Table 3. MVd of the whole study population increased over time, 0.19 ± 0.06 at baseline, 0.41 ± 0.17 at 1 min, 0.46 ± 0.13 at 2 min, and 0.47 ± 0.13 m/s at 3 min, resulting in sequential increase in CFvR, 2.20 ± 0.87 at 1 min, 2.47 ± 0.77 at 2 min, and 2.53 ± 0.72 at 3 min.

**Table 3:** Echocardiographic coronary blood flow velocity parameters

	Total patients (N = 202)	Men (N = 64)	Women (N = 138)	*P*-value
Baseline coronary flow velocity				
Systolic blood pressure (mmHg)	137.2 ± 16.9	133.2 ± 14.7	139.1 ± 17.6	0.021
Diastolic blood pressure (mmHg)	80.8 ± 11.5	82.4 ± 10.9	80.1 ± 11.7	0.195
Heart rate (bpm)	65.0 ± 11.0	62.5 ± 10.4	66.1 ± 11.1	0.028
Rate pressure product (bpm × mmHg)	8520.0 (7482.0–10005.0)	8175.0 (7473.0–9027.0)	8925.0 (7494.0–10289.5)	0.007
Peak diastolic velocity (m/s)	0.26 ± 0.09	0.26 ± 0.08	0.25 ± 0.07	0.192
Diastolic deceleration time (ms)	969.5 (708.0–1232.0)	1059.0 (837.5–1307.5)	925.5 (657.0–1191.0)	0.007
Mean diastolic velocity (m/s)	0.19 ± 0.06	0.20 ± 0.06	0.18 ± 0.05	0.071
Peak systolic velocity (m/s)	0.14 ± 0.05	0.14 ± 0.05	0.14 ± 0.05	0.495
Mean systolic velocity (m/s)	0.11 ± 0.04	0.11 ± 0.04	0.11 ± 0.03	0.772
Peak diastolic velocity (m/s)	0.26 ± 0.09	0.26 ± 0.08	0.25 ± 0.07	0.192
Diastolic deceleration time (ms)	969.5 (708.0–1232.0)	1059.0 (837.5–1307.5)	925.5 (657.0–1191.0)	0.007
Mean diastolic velocity (m/s)	0.19 ± 0.06	0.20 ± 0.06	0.18 ± 0.05	0.071
Peak diastolic-systolic velocity ratio	1.91 ± 0.41	1.96 ± 0.40	1.89 ± 0.41	0.272
Mean diastolic-systolic velocity ratio	1.76 ± 0.38	1.79 ± 0.36	1.75 ± 0.39	0.463
Coronary flow velocity at 1 min after adenosine infusion				
Heart rate (bpm)	74.1 ± 14.9	72.0 ± 14.6	75.0 ± 15.0	0.205
Peak diastolic velocity (m/s)	0.58 ± 0.25	0.65 ± 0.25	0.55 ± 0.24	0.008
Diastolic deceleration time (ms)	709.5 (515.0–980.0)	709.5 (560.5–968.5)	705.5 (513.0–981.0)	0.745
Mean diastolic velocity (m/s)	0.41 ± 0.17	0.45 ± 0.17	0.39 ± 0.17	0.021
Peak systolic velocity (m/s)	0.32 ± 0.15	0.34 ± 0.15	0.31 ± 0.15	0.295
Mean systolic velocity (m/s)	0.25 ± 0.11	0.26 ± 0.11	0.24 ± 0.11	0.287
Peak diastolic–systolic velocity ratio	1.89 ± 0.44	2.03 ± 0.49	1.83 ± 0.40	0.003
Mean diastolic–systolic velocity ratio	1.74 ± 0.41	1.83 ± 0.45	1.69 ± 0.39	0.035
CFvR by peak diastolic velocity at 1 min	2.29 ± 0.96	2.60 ± 0.97	2.15 ± 0.92	0.002
CFvR by mean diastolic velocity at 1 min	2.20 ± 0.87	2.44 ± 0.87	2.09 ± 0.86	0.008
Coronary flow velocity at 2 min after adenosine infusion				
Heart rate (bpm)	78.28 ± 14.64	76.0 ± 14.1	79.3 ± 14.8	0.405
Peak diastolic velocity (m/s)	0.65 ± 0.21	0.70 ± 0.24	0.63 ± 0.19	0.033
Diastolic deceleration time (ms)	601.0 (446.5–797.0)	604.0 (489.0–794.0)	598.0 (420.0–800.0)	0.530
Mean diastolic velocity (m/s)	0.46 ± 0.13	0.49 ± 0.15	0.44 ± 0.12	0.039
Peak systolic velocity (m/s)	0.36 ± 0.13	0.36 ± 0.15	0.36 ± 0.12	0.841
Mean systolic velocity (m/s)	0.28 ± 0.10	0.28 ± 0.11	0.28 ± 0.10	0.917
Peak diastolic-systolic velocity ratio	1.89 ± 0.48	2.06 ± 0.64	1.81 ± 0.35	0.005
Mean diastolic-systolic velocity ratio	1.71 ± 0.42	1.83 ± 0.55	1.65 ± 0.33	0.014
CFvR by peak diastolic velocity at 2 min	2.59 ± 0.85	2.82 ± 0.96	2.49 ± 0.78	0.021
CFvR by mean diastolic velocity at 2 min	2.47 ± 0.77	2.63 ± 0.85	2.39 ± 0.72	0.046
Coronary flow velocity at 3 min (peak) after adenosine infusion				
Systolic blood pressure (mmHg)	130.6 ± 16.7	131.6 ± 15.6	129.7 ± 17.2	0.462
Diastolic blood pressure (mmHg)	76.0 ± 13.0	80.0 ± 12.9	74.1 ± 12.7	0.003
Heart rate (bpm)	81.3 ± 14.9	77.8 ± 13.9	82.9 ± 15.1	0.022
Rate pressure product (bpm × mmHg)	10,455.0 (8832.0–12,464.0)	10,097.5 (8820.0–12,319.0)	10,530.0 (8909.0–12,537.0)	0.360
Rate pressure product reserve	1661.9 ± 1537.8	1954.5 ± 1447.2	1523.5 ± 1565.1	0.069
Chest pain after adenosine infusion, n (%)	14 (7.1)	6 (9.5)	8 (6.0)	0.543
Peak diastolic velocity (m/s)	0.67 ± 0.20	0.72 ± 0.22	0.65 ± 0.18	0.013
Diastolic deceleration time (ms)	531.0 (401.0–733.0)	599.0 (425.5–796.0)	512.0 (376.0–721.0)	0.076
Peak systolic velocity (m/s)	0.38 ± 0.13	0.39 ± 0.15	0.38 ± 0.12	0.570
Peak diastolic–systolic velocity ratio	1.82 ± 0.37	1.96 ± 0.47	1.76 ± 0.29	0.002
Mean diastolic velocity (m/s)	0.47 ± 0.13	0.50 ± 0.14	0.46 ± 0.13	0.020
Mean systolic velocity (m/s)	0.30 ± 0.10	0.30 ± 0.11	0.29 ± 0.09	0.573
Mean diastolic-systolic velocity ratio	1.64 ± 0.35	1.76 ± 0.45	1.59 ± 0.29	0.009
CFvR by peak diastolic velocity	2.68 ± 0.81	2.88 ± 0.85	2.59 ± 0.77	0.016
CFvR by mean diastolic velocity	2.53 ± 0.72	2.68 ± 0.73	2.45 ± 0.70	0.033
Coronary microvascular dysfunction	84 (41.6)	20 (31.2)	64 (46.4)	0.061

*CFvR* coronary flow velocity reserve

Women exhibited higher RPP at baseline (8925.0 [IQR 7494.0–10289.5] vs 8175.0 [IQR 7473.0–9027.0] beats/min x mmHg; p = 0.007). At baseline, no difference was seen in peak diastolic velocity between women and men (0.25 ± 0.07 vs 0.26 ± 0.08 m/s, p = 0.192). But MVd was lower in women than in men with statistical trend (0.18 ± 0.05 vs 0.20 ± 0.06 m/s, p = 0.071). The mean systolic velocity (0.11 ± 0.03 vs 0.11 ± 0.04 m/s; p = 0.772) and mean diastolic-systolic velocity ratio (1.75 ± 0.39 vs 1.79 ± 0.36; p = 0.463) of CBFs were similar between women and men. Diastolic deceleration time of CBF was shorter in women than in men.

**Fig. 2:**
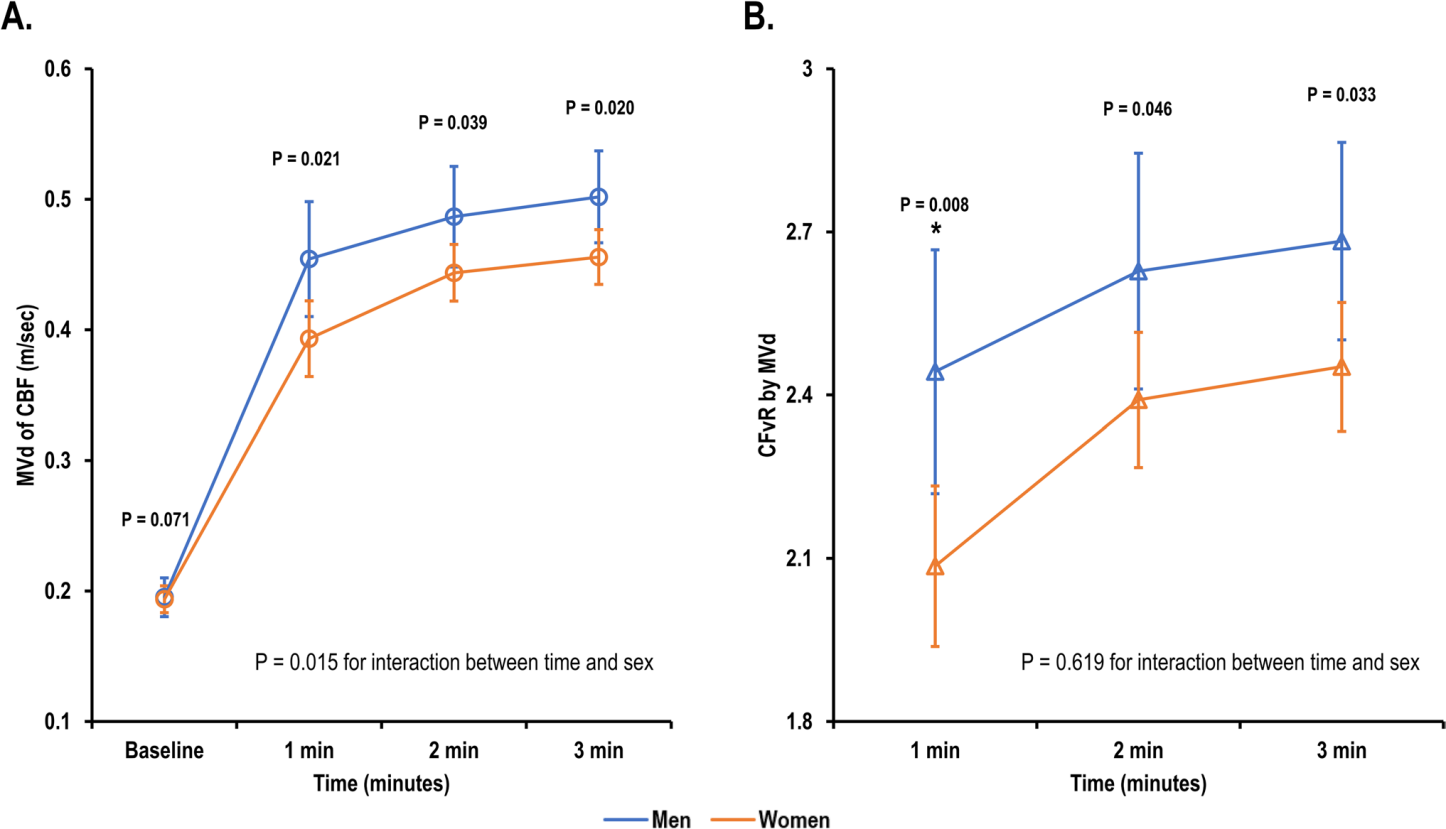
Sex difference in serial changes in CBF velocities over time after adenosine infusion. **A** Serial changes in MVd of CBF over time. **B** Serial changes in CFvR by MVd over time. * indicates corrected p < 0.0125 for MVd of CBF or < 0.0166 for CFvR by Bonferroni’s post-hoc analysis. *CBF* coronary blood flow, *CFvR* coronary flow velocity reserve, *MVd* mean diastolic velocity of coronary flow

At peak stress with adenosine, RPP became comparable between the sexes (10,530.0 [IQR 8909.0–12537.0] vs 10,097.5 [IQR 8820.0–12319.0] beats/min × mmHg; p = 0.360). However, the sex difference in CBF was prominent during serial evaluation at 1 min. At 1 min, the peak diastolic velocity and MVd in women were lower than those of men, and these differences continued until 3 min of adenosine infusion (MVd at 1 min, 0.39 ± 0.17 vs 0.45 ± 0.17 m/s; 2 min, 0.44 ± 0.12 vs 0.49 ± 0.15 m/s; 3 min, 0.46 ± 0.13 vs 0.50 ± 0.14, p = 0.015 for interaction between time and sex, Fig. 2A). This resulted in lower CFvR in women than in men at 1 min (2.09 ± 0.86 vs 2.44 ± 0.87; p = 0.008), 2 min (2.39 ± 0.72 vs 2.63 ± 0.85; p = 0.046), and 3 min (2.45 ± 0.70 vs 2.68 ± 0.73; p = 0.033) (Fig. 2B). In women, the predominant increase in MVd was observed at 1 min after adenosine administration, with no significant further increase observed thereafter.

**Fig. 3:**
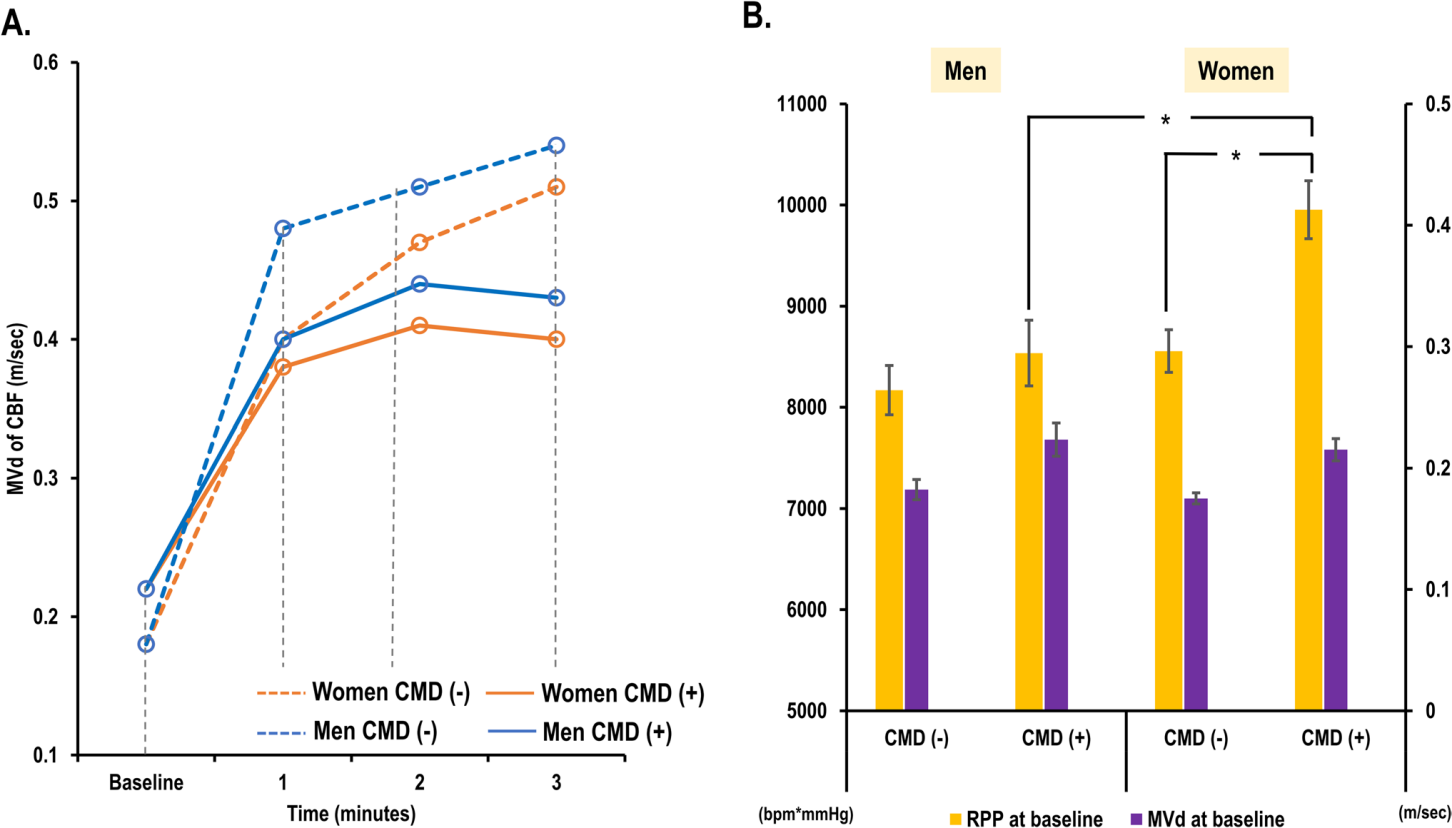
CBF velocities and RPP according to sex or the presence of CMD. **A** Serial changes in MVd of CBF over time. Men with CMD exhibited similar patterns of serial changes in CBF velocities compared to women with CMD. **B** RPP and MVd of CBF according to sex or the presence of CMD. *CMD* coronary microvascular dysfunction, *CBF* coronary blood flow, *CMD* coronary microvascular dysfunction, *MVd* mean diastolic velocity of coronary flow, *RPP* rate pressure product

CMD was present in approximately 40% of the study population with a higher prevalence among women (n = 64 [46.4%] vs n = 20 [31.2%], p = 0.06). Patients with CMD exhibited a higher incidence of hypertension and diabetes, as well as elevated baseline RPP in comparison to those without CMD (Supplementary Table 1). Upon stratification by sex, no disparities in demographic characteristics emerged with regard to the presence of CMD in both men and women, respectively (Supplementary Table 2). Baseline MVd of CBF and RPP was higher in patients with CMD, regardless of sex (Supplementary Table 3). Men with CMD exhibited similar patterns of serial changes in CBF compared to women with CMD (Fig. 3A), with higher RPP at baseline in women with CMD (9792.0 [IQR 8260.0–11325.0] vs 8430.0 [IQR 7922.0–9246.0] beats/min x mmHg; p = 0.012) (Fig. 3B). In the univariable analysis, female, hypertension, diabetes, dyslipidemia, and a high baseline RPP were the predictors for the presence of CMD. Among the echocardiographic parameters, only E/e′ at baseline exhibited a weak correlation with CFvR (r = − 0.161, p = 0.023). However, no echocardiographic parameter emerged as a predictor for the presence of CMD in univariable analysis. In the multivariable analysis, baseline RPP remained as an independent predictor for CMD (Supplementary Table 4).

### Association between exercise capacity and coronary flow velocity reserve

Women exhibited shorter duration of exercise time than men (8′ 56″ (IQR 6′ 44″–10′ 4″] vs 10′ 2″ [IQR 8′ 14″–11′ 54″], p = 0.001), which resulted in a lower Duke score (7 (IQR 2.75–9] vs 9 [IQR 6–11]; p < 0.001) as well as a lower amount of work (10.2 [IQR 7.8–11.4] vs 10.7 [IQR 10.0–13.3] metabolic equivalent of task; p = 0.011).

Total exercise time, amount of work, and Duke score were directly related to CFvR (Supplementary Fig. 1), especially in men (total exercise time, r = 0.326, p = 0.018; amount of work, r = 0.368, p = 0.007; Duke score, r = 0.327, p = 0.018) (Supplementary Fig. 2). Women with CMD were more likely to exhibit horizontal or downsloping ST depression during the treadmill test compared to men with CMD (n = 12 [25.5%] vs n = 3 [18.8%]; p = 0.833).

### Clinical outcomes

Throughout the 1-year follow-up period, there were no reported fatalities among the patients. However, three patients were diagnosed with stroke incidents. Patient 1, a 44-year-old woman, presented with severe headache and was diagnosed with lacunar infarction in the left basal ganglia after 15 days of follow-up. Patient 2, a 69-year-old woman, developed a sudden headache at 173 days of follow-up; further assessment revealed significant stenosis in the left middle cerebral artery through brain magnetic resonance angiography. Patient 3, a 59-year-old woman, was diagnosed with focal cerebral infarction at right temporal and parietal lobe at 270 days of follow-up. No significant differences in CBF parameters were observed between patients with event and those without (CFvR 2.55 (2.12–2.64) vs. 2.44 (2.04–2.88), p = 0.746). Supplementary Table 5 provides a summary of the demographic and echocardiographic data for patients with event.

## Discussion

The present study sheds light on the potential differences in coronary microvascular function between men and women with suspected angina but with no obstructive coronary arteries. Women had lower CBF velocities and CFvR than men, even when they exhibited higher LVEF and GLS. The prevalence of CMD was higher among women using a criterion of CFvR < 2.3. Women also exhibited slower and gradual increases in CBF velocities during serial evaluation after adenosine infusion. Conversely, men exhibited faster and steeper increases in CBF velocities. Exercise capacity measured using the treadmill exercise test was directly related to CFvR, especially among men.

To the best of our knowledge, this is the first study to evaluate the immediate response of CBF serially after adenosine infusion and possible sex differences. Our results showed that women exhibited slower and low-intensity increases in CBF velocities during serial evaluation after adenosine infusion, even though there was no significant sex difference in the resting CBF velocities. Additionally, women with CMD had higher baseline RPP, indicating higher oxygen consumption, compared to men with CMD. Women typically have smaller cardiac chambers and lower stroke volume compared to men, which may lead to adaptations such as higher LVEF, GLS, and heart rate to maintain adequate cardiac output [18]. Therefore, women demonstrate higher oxygen consumption [19], a finding consistent with the observations of the present study. While these adaptations may confer some benefits, they may also contribute to increased susceptibility to myocardial ischemia and heart failure with the preserved ejection fraction (HFpEF) in a condition that deteriorates coronary vascular function and causes coronary vascular obstruction in women.

Women exhibited smaller epicardial coronary arteries than men, even considering body size and LV mass, and higher CBF at rest and in hyperemic status [20]. Coronary arteries of women are smaller in diameter but higher in flow, exhibiting higher endothelial shear stress (ESS) and developing laminar flow. This results in sex difference in susceptibility of coronary artery disease [21]. The ESS refers to the frictional force exerted by blood flow on the endothelial cells that line the blood vessel wall. When ESS is low, blood flow is disrupted and is turbulent, leading to a range of detrimental effects, like atherosclerosis [22] and inflammation [23]. ESS generally decreases with age, although at a faster rate in women than in men, especially in those in the late 50 s to early 60 s [24]. This is a critical age range for women, as arterial stiffening typically begins at this time, and the decrease in ESS may exacerbate this problem. Further, menopause affects arterial elasticity due to a decrease in estrogen levels, which could be related to the decline in ESS. The low ESS also affects the occurrence of CMD, and the two aggravate each other. In the present study, women with suspected angina but non-obstructed coronary arteries had lower CBF even in hyperemic condition. Lower CBF in women, which might have resulted in lower ESS. Therefore, this can explain and demonstrate the higher prevalence of CMD in women. Lower CBF and ESS could explain the higher prevalence of INOCA among women.

Furthermore, we observed that women exhibited higher levels of NT-proBNP and baseline E/e′ compared to men. These findings are aligned with the previous epidemiological data that demonstrate the predisposition of women to HFpEF [25]. Considering the high prevalence of CMD in patients with HFpEF [6], sex difference in the prevalence of HFpEF may contribute to the increased prevalence of CMD among women. It’s worth noting that this disparity could be linked to a more gradual and slower increase in CFvR in women compared to men.

Interestingly, our study also revealed that exercise capacity measured using the treadmill exercise test was directly related to CFvR, especially among men. This finding conflicts with previous studies [8], and suggests that the relationship between exercise capacity and coronary microvascular function may differ between men and women. Slower and gradual increases in CBF may contribute to myocardial ischemia at an early stage of exercise, which may result in reduced exercise capacity. Additionally, dyspnea due to higher E/e′ and possible peripheral muscle weakness in women might play a role in this mechanism. In a future study, we aim to directly measure maximal oxygen consumption according to cardiopulmonary exercise capacity and sarcopenia using a body composition analysis to determine the effect of peripheral muscle weakness and CMD on exercise capacity in patients with suspected angina and non-obstructed coronary arteries [26]. Remarkably, only one-fifth of men with CMD and one-fourth of women with CMD exhibited horizontal or downsloping ST depression during the treadmill test. The sensitivity and specificity of a positive treadmill test were found to be 23.8% and 75.8%, respectively. This observation aligns with recent research suggesting that conventional exercise tests may be insufficient for assessing inducible ischemia in patients with CMD [27].

The present study had several limitations. Firstly, it exclusively assessed endothelial-independent coronary microvascular function, leading to a lower CMD prevalence compared to a prior study [28]. An echocardiographic measurement of CFvR might be less accurate than an invasive measurement of CFvR. However, CFvR measured by echocardiography was closely correlated with CFvR measured by angiography [29]. Consequently, the limitations associated with CFvR assessment via echocardiography appear to be minimal. Secondly, twice as many women were enrolled in the present study compared to men. This divergence in enrollment may reflect real-world trends, as women presenting with suspected angina are more likely to exhibit INOCA. Thirdly, the treadmill exercise test aimed for maximal tolerable exertion, potentially influencing exercise capacity due to orthopedic issues or subjective fatigue. Fourthly, this study solely encompassed 1-year follow-up outcomes, and the low event rate hindered the demonstration of significant findings, thereby limiting insights into the prognostic implications of the results. Consequently, further investigations necessitate larger participant cohorts, more objective assessments of exercise capacity, and long-term follow-up data.

## Conclusion

In patients with suspected angina but with no obstructive coronary arteries, CMD was especially prevalent among women. Women had higher oxygen consumption levels, even with similar LV mass index at baseline while exhibiting slower and gradual increases in CBF velocities during adenosine stress echocardiography. In contrast, men exhibited faster and steeper increases in CBF velocities even with similar RPP in stress. Higher oxygen consumption and slower and gradual increases in CBF velocities may contribute to the greater vulnerability of women to ischemic insults and angina symptoms compared to men.

## Perspectives and significance

The study sheds light on the potential differences in coronary microvascular function between men and women with suspected angina and no obstructed coronary arteries. Women with suspected angina had lower coronary flow velocity reserve than men, even when they had higher baseline oxygen consumption, left ventricular ejection fraction, and global longitudinal strain. Women showed higher prevalence of coronary microvascular dysfunction than men, accordingly. During serial evaluation after adenosine infusion, women exhibited slower and gradual increases in coronary blood flow velocities than men, even with similar oxygen consumption in stress between women and men. Relatively smaller coronary arteries but lower coronary blood flow velocities even in hyperemic condition might have resulted in lower endothelial shear stress with turbulent flow than men. It can contribute to the greater vulnerability of women to ischemic insults and angina symptoms, and higher prevalence of coronary microvascular dysfunction compared to men. This study highlights the importance of considering sex-specific differences in the evaluation of coronary microvascular function, as these differences could have implications for the interpretation of diagnostic tests and the selection of treatment strategies.

## Supplementary Information

Below is the link to the electronic supplementary material.

Supplementary file1 (DOCX 1319 kb)

## Data Availability

The datasets used and analyzed during the current study are available from the corresponding author on reasonable request.
