# Supplementary material for: Sex differences of sequential changes in coronary blood flow and microvascular function in patients with suspected angina
Source: Clin Res Cardiol. 2023 Dec 19;113(12):1638–49. doi: 10.1007/s00392-023-02358-2 (PMC11579202; doi:10.1007/s00392-023-02358-2)
Supplement: Supplementary file 1 — Supplementary file1 (DOCX 1319 kb) [file 392_2023_2358_MOESM1_ESM.docx]

**Sex differences of sequential changes in coronary blood flow and microvascular function in patients with suspected angina**

So Ree Kim, Mi-Na Kim, Dong-Hyuk Cho, Hee-Dong Kim, Sung A Bae, Hack-Lyoung Kim, Myung-A Kim,

Kyung-Soon Hong, Wan Joo Shim, and Seong-Mi Park

**Table of Contents**

**(1) Supplementary Tables**

**Supplementary Table 1. Baseline demographics and echocardiographic parameters according to the presence of CMD**

**Supplementary Table 2. Baseline demographics and echocardiographic parameters according to sex or the presence of CMD**

**Supplementary Table 3. Echocardiographic coronary blood flow velocity parameters and data from treadmill exercise test according to sex or the presence of CMD**

**Supplementary Table 4. Independent predictors of the presence of CMD**

**Supplementary Table 5. Demographic and echocardiographic data of patients with event**

**(2) Supplementary Figure and Figure Legend**

**Supplementary Figure 1. Correlation between CFR by MVd and exercise capacity**

**Supplementary Figure 2. Correlation between CFR by MVd and exercise capacity according to sex**

**(1) Supplementary Tables**

**Supplementary Table 1. Baseline demographics and echocardiographic parameters according to the presence of CMD**

|  | **CMD- (N = 118)** | **CMD+ (N = 84)** | ***P*-value** |
| --- | --- | --- | --- |
| Age (years) | 60 (57 – 67) | 62.5 (54 – 70) | 0.529 |
| Women | 74 (62.7) | 64 (76.2) | 0.061 |
| Body surface area (m^2^) | 1.70 ± 0.17 | 1.66 ± 0.16 | 0.110 |
| Body mass index (kg/m^2^) | 25.2 ± 2.4 | 25.2 ± 2.9 | 0.969 |
| Hypertension, n (%) | 52 (44.1) | 53 (63.9) | 0.009 |
| Diabetes, n (%) | 9 (7.6) | 15 (18.1) | 0.043 |
| Dyslipidemia, n (%) | 48 (40.7) | 47 (56.6) | 0.080 |
| Current or Ex-smoker, n (%) | 26 (22.0) | 11 (13.2) | 0.051 |
| eGFR (ml/min/1.73m^2^) | 86.1 (75.8 – 97.6) | 84.2 (72.5 – 99.3) | 0.613 |
| NT-pro BNP (pg/mL) | 36.8 (21.1 – 79.7) | 53.2 (22.1 – 94.0) | 0.338 |
| LV mass index (g/m^2^) | 81.6 (75.3 – 93.8) | 78.9 (67.2 – 91.6) | 0.046 |
| Relative wall thickness | 0.39 (0.36 – 0.42) | 0.38 (0.35 – 0.43) | 0.559 |
| LV ejection fraction (%) at baseline | 62.2 ± 5.7 | 62.8 ± 5.7 | 0.468 |
| Left atrial volume index (ml/m^2^) at baseline | 26.0 (21.4 – 34.8) | 27.2 (22.0 – 33.3) | 0.819 |
| Mitral E velocity (cm/s) at baseline | 57.3 (47.0 – 70.0) | 61.0 (50.0 – 76.6) | 0.060 |
| Medial e’ velocity (cm/s) at baseline | 6.4 ± 1.7 | 6.5 ± 1.8 | 0.675 |
| Medial E/e’ at baseline | 9.1 (7.6 – 11.2) | 9.7 (8.2 – 11.9) | 0.122 |
| Global longitudinal strain (%) at baseline | -19.3 ± 2.5 | -19.8 ± 2.6 | 0.197 |
| RPP (bpm x mmHg) at baseline | 8128 (7290 – 9516) | 9230 (8125 – 10725) | <0.001 |
| RPP (bpm x mmHg) at peak | 10107.1 ± 2049.0 | 11251.5 ± 2678.0 | 0.002 |
| MVd (m/sec) at baseline | 0.17 (0.15 – 0.21) | 0.20 (0.17 – 0.25) | <0.001 |
| MVd (m/sec) at 1 minute | 0.45 (0.30 – 0.53) | 0.39 (0.27 – 0.47) | 0.066 |
| MVd (m/sec) at 2 minutes | 0.48 (0.41 – 0.55) | 0.42 (0.34 – 0.49) | 0.001 |
| MVd (m/sec) at peak | 0.50 (0.43 – 0.60) | 0.38 (0.33 – 0.48) | <0.001 |
| CFR by MVd at 1 minute | 2.69 (1.90 – 3.13) | 1.91 (1.15 – 2.18) | <0.001 |
| CFR by MVd at 2 minutes | 2.71 (2.42 – 3.23) | 2.00 (1.69 – 2.17) | <0.001 |
| CFR by MVd at peak | 2.76 (2.53 – 3.33) | 1.94 (1.75 – 2.10) | <0.001 |
| LV ejection fraction (%) at peak | 69.0 (64.7 – 72.6) | 70.4 (65.1 – 73.2) | 0.767 |
| Left atrial volume index (ml/m^2^) at peak | 29.7 (23.4 – 35.7) | 29.6 (24.1 – 38.9) | 0.947 |
| Mitral E velocity (cm/s) at peak | 78.2 (67.3 – 89.1) | 83.1 (72.7 – 94.0) | 0.051 |
| Medial e’ velocity (cm/s) at peak | 7.1 (6.0 – 8.4) | 7.5 (6.0 – 8.8) | 0.561 |
| Diastolic reserve (cm/s) | 0.70 (-0.23 – 1.70] | 0.67 (-0.10 – 2.15) | 0.543 |
| Medial E/e’ at peak | 10.9 (9.3 – 12.4) | 11.0 (9.0 – 13.7) | 0.704 |
| Global longitudinal strain (%) at peak | -21.8 ± 2.6 | -22.1 ± 2.5 | 0.337 |
| %Change of Global longitudinal strain (%) | 11.3 (6.6 – 18.1) | 12.4 (6.2 – 19.0) | 0.958 |

Abbreviations: CFR, coronary flow velocity reserve; CMD, coronary microvascular dysfunction; E, early diastolic mitral inflow velocity; e’, early diastolic mitral annulus velocity; eGFR, estimated glomerular filtration rate; LV, left ventricular; MVd, mean diastolic velocity of coronary flow; NT-proBNP, N-terminal pro-B-type natriuretic peptide, RPP, rate pressure product.

Diastolic reserve was defined as peak e’ – baseline e’.

**Supplementary Table 2. Baseline demographics and echocardiographic parameters according to sex or the presence of CMD**

|  | **Men (N = 64)** | | |  | **Women (N = 138)** | | |
| --- | --- | --- | --- | --- | --- | --- | --- |
|  | **CMD-**  **(N = 44)** | **CMD+**  **(N = 20)** | ***P*-value** |  | **CMD-**  **(N = 74)** | **CMD+**  **(N = 64)** | ***P*-value** |
| Age (years) | 59.8 ± 9.5 | 61.5 ± 11.2 | 0.544 |  | 61.7 ± 8.5 | 61.7 ± 10.7 | 0.994 |
| Body surface area (m^2^) | 1.84 ± 0.13 | 1.82 ± 0.12 | 0.714 |  | 1.61 ± 0.13 | 1.61 ± 0.13 | 0.775 |
| Body mass index (kg/m^2^) | 25.2 ± 2.4 | 25.2 ± 2.9 | 0.969 |  | 25.0 ± 3.7 | 24.8 ± 3.1 | 0.724 |
| Hypertension, n (%) | 18 (40.9) | 12 (60.0) | 0.251 |  | 33 (44.6) | 34 (54.0) | 0.356 |
| Diabetes, n (%) | 4 (9.1) | 4 (20.0) | 0.415 |  | 5 (6.8) | 11 (17.5) | 0.093 |
| Dyslipidemia, n (%) | 21 (47.7) | 11 (55.0) | 0.470 |  | 27 (36.5) | 36 (57.1) | 0.050 |
| Current or Ex-smoker, n (%) | 22 (50.0) | 7 (35.0) | 0.230 |  | 4 (5.4) | 4 (6.4) | 0.092 |
| NT-pro BNP (pg/mL) | 22.5 (11.8-42.8) | 23.0 (16.2-53.2) | 0.694 |  | 44.1 (25.8-118.3) | 63.1 (31.7-94.1) | 0.996 |
| LV ejection fraction (%) at baseline | 61.75 ± 5.36 | 58.48 ± 4.82 | 0.023 |  | 62.48 ± 5.94 | 64.16 ± 5.32 | 0.086 |
| Left atrial volume index (ml/m^2^) at baseline | 25.5 (22.3-32.8) | 25.5 (20.8-31.2) | 0.750 |  | 26.8 (20.9-36.5) | 27.3 (22.4-33.6) | 0.784 |
| Mitral E velocity (cm/s) at baseline | 51.0 (44.0-64.0) | 50.0 (45.5-59.6) | 0.885 |  | 59.0 (48.0-73.7) | 65.0 (55.2-77.5) | 0.070 |
| Medial e’ velocity (cm/s) at baseline | 6.2 ± 1.6 | 6.2 ± 1.6 | 0.932 |  | 6.4 ± 1.8 | 6.6 ± 1.9 | 0.715 |
| Medial E/e’ at baseline | 8.9 (7.1-11.0) | 9.0 (7.0-10.8) | 0.971 |  | 9.4 (7.7-11.2) | 9.9 (8.6-12.2) | 0.132 |
| Global longitudinal strain (%) at baseline | -18.7 ± 2.6 | -17.4 ± 1.8 | 0.049 |  | -19.7 ± 2.3 | -20.6 ± 2.4 | 0.041 |
| LV ejection fraction (%) at peak | 67.6 ± 5.3 | 63.7 ± 6.8 | 0.015 |  | 69.9 ± 5.2 | 70.5 ± 5.4 | 0.498 |
| Left atrial volume index (ml/m^2^) at peak | 30.5 (24.5-35.5) | 25.7 (22.1-32.5) | 0.368 |  | 29.0 (23.4-35.7) | 30.2 (25.0-39.9) | 0.546 |
| Mitral E velocity (cm/s) at peak | 74.5 (62.1-84.6) | 78.8 (72.3-88.2) | 0.391 |  | 81.1 (69.2-94.0) | 87.5 (73.4-99.7) | 0.106 |
| Medial e’ velocity (cm/s) at peak | 7.3 (6.2-8.2) | 6.9 (6.0-8.2) | 0.479 |  | 7.0 (5.7-8.5) | 7.8 (6.1-8.9) | 0.277 |
| Diastolic reserve (cm/s) | 0.80 (-0.10-1.80) | 0.70 (-0.13-1.21) | 0.628 |  | 0.67 (-0.30-1.60) | 0.67 (0.05-2.40) | 0.266 |
| Medial E/e’ at peak | 10.4 (8.9-11.9) | 11.0 (8.9-12.4) | 0.606 |  | 11.2 (9.6-13.6) | 11.0 (9.1-14.0) | 0.915 |
| Global longitudinal strain (%) at peak | -21.1 ± 2.3 | -19.9 ± 1.7 | 0.036 |  | -22.2 ± 2.7 | -22.9 ± 2.2 | 0.097 |
| %Change of Global longitudinal strain (%) | 11.6 (6.9-18.2) | 10.5 (6.4-21.4) | 0.883 |  | 11.1 (6.8-17.0) | 12.4 (6.4-18.9) | 1.000 |

Abbreviations: CMD, coronary microvascular dysfunction; E, early diastolic mitral inflow velocity; e’, early diastolic mitral annulus velocity; LV, left ventricular; NT-proBNP, N-terminal pro-B-type natriuretic peptide.

Diastolic reserve was defined as peak e’ – baseline e’.

**Supplementary Table 3. Echocardiographic coronary blood flow velocity parameters and data from treadmill exercise test according to sex or the presence of CMD**

|  | **Men (N = 64)** | | |  | **Women (N = 138)** | | |
| --- | --- | --- | --- | --- | --- | --- | --- |
|  | **CMD-**  **(N = 44)** | **CMD+**  **(N = 20)** | ***P*-value** |  | **CMD-**  **(N = 74)** | **CMD+**  **(N = 64)** | ***P*-value** |
| RPP (bpm x mmHg) at baseline | 7936.5 (6912.0-9027.0) | 8430.0 (7922.0-9246.0) | 0.148 |  | 8215.0 (7317.5-9683.0) | 9792.0 (8260.0-11325.0) | <0.001 |
| RPP (bpm x mmHg) at peak | 10425.0 (8832.0-11875.0) | 9728.5 (8410.0-12391.5) | 0.857 |  | 9625.0 (8558.0-11133.0) | 11620.0 (9383.0-13353.5) | <0.001 |
| MVd (m/sec) at baseline | 0.18 ± 0.06 | 0.22 ± 0.06 | 0.009 |  | 0.18 ± 0.04 | 0.22 ± 0.07 | <0.001 |
| MVd (m/sec) at 1 minute | 0.48 ± 0.17 | 0.40 ± 0.17 | 0.088 |  | 0.40 ± 0.18 | 0.38 ± 0.15 | 0.443 |
| MVd (m/sec) at 2 minutes | 0.51 ± 0.15 | 0.44 ± 0.14 | 0.108 |  | 0.47 ± 0.12 | 0.41 ± 0.13 | 0.015 |
| MVd (m/sec) at peak | 0.54 ± 0.14 | 0.43 ± 0.11 | 0.004 |  | 0.51 ± 0.11 | 0.40 ± 0.12 | <0.001 |
| CFR by MVd at 1 minute | 2.74 ± 0.78 | 1.80 ± 0.68 | <0.001 |  | 2.35 ± 0.98 | 1.78 ± 0.55 | <0.001 |
| CFR by MVd at 2 minutes | 2.92 ± 0.79 | 1.99 ± 0.59 | <0.001 |  | 2.77 ± 0.71 | 1.95 ± 0.41 | <0.001 |
| CFR by MVd at peak | 3.02 ± 0.60 | 1.94 ± 0.28 | <0.001 |  | 2.95 ± 0.55 | 1.87 ± 0.28 | <0.001 |
| Total exercise time (seconds) | 626.0 (544.0-720.0) | 518.0 (316.0-649.5) | 0.068 |  | 535.5 (403.5-626.0) | 541.0 (411.0-582.0) | 1.000 |
| Amount of work (metabolic equivalent of task) | 11.5 (10.0-13.5) | 10.0 (6.9-12.6) | 0.048 |  | 10.1 (7.8-12.0) | 10.2 (8.1-11.1) | 0.904 |
| Duke treadmill score | 10.0 (8.0-11.0) | 6.0 (3.5-8.5) | 0.024 |  | 6.0 (3.0-9.0) | 8.0 (2.5-9.0) | 0.733 |
| Presence of ST depression during treadmill exercise test | 8 (21.6) | 3 (20.0) | 1.000 |  | 16 (26.7) | 12 (26.7) | 1.000 |

Abbreviations: CFR, coronary flow velocity reserve; CMD, coronary microvascular dysfunction; MVd, mean diastolic velocity of coronary flow; RPP, rate pressure product.

**Supplementary Table 4. Independent predictors of the presence of CMD**

| **Variables** | **Univariable** | | **Multivariable** | |
| --- | --- | --- | --- | --- |
|  | **Odds ratio (95% CI)** | ***P*** | **Odds ratio (95% CI)** | ***P*** |
| Age | 1.01 (0.98 – 1.04) | 0.634 | 0.98 (0.94 – 1.02) | 0.349 |
| Female | 1.90 (1.03 – 3.61) | 0.044* | 0.91 (1.53 – 2.39) | 0.835 |
| Body mass index | 0.98 (0.90 – 1.07) | 0.689 | 0.95 (0.85 – 1.06) | 0.397 |
| Hypertension | 2.24 (1.27 – 4.02) | 0.006* | 1.65 (0.76 – 3.64) | 0.209 |
| Diabetes | 2.67 (1.13 – 6.68) | 0.029* | 2.14 (0.74 – 6.36) | 0.162 |
| Dyslipidemia | 1.87 (1.05 – 3.37) | 0.035* | 1.46 (0.72 – 2.97) | 0.291 |
| Current smoker | 0.56 (0.31 – 0.94) | 0.038* | 060 (0.29 – 1.16) | 0.145 |
| LV mass index at baseline | 0.98 (0.96 – 1.00) | 0.059 | 0.98 (0.95 – 1.00) | 0.063 |
| E/e’ at baseline | 1.06 (0.96 – 1.16) | 0.245 | 1.06 (0.94 – 1.20) | 0.304 |
| Global longitudinal strain at baseline | 0.93 (0.83 – 1.04) | 0.197 | 0.92 (0.79 – 1.07) | 0.290 |
| RPP (bpm x mmHg) at baseline | 1.0003 (1.0002 – 1.0005) | <0.001* | 1.0003 (1.0001 – 1.0005) | 0.003 |

Abbreviations: CI, confidence interval; CMD, coronary microvascular dysfunction; E, early diastolic mitral inflow velocity; e’, early diastolic mitral annulus velocity; LV, left ventricular; RPP, rate pressure product

**Supplementary Table 5. Demographic and echocardiographic data of patients with event**

|  | **Age** | **Sex** | **BMI (kg/m2)** | **Hypertension** | **Diabetes** | **Smoking** | **LVEF (%)**  **at baseline** | **E/e’**  **at baseline** | **GLS (%)**  **at baseline** | **CFR** | **Time to event (days)** | **Event** |
| --- | --- | --- | --- | --- | --- | --- | --- | --- | --- | --- | --- | --- |
| Patient 1 | 44 | female | 20.2 | No | No | Never | 70.6 | 8.9 | -21.9 | 2.74 | 15 | lacunar infarction at left basal ganglia |
| Patient 2 | 69 | female | 23.3 | Yes | Yes | Never | 71.7 | 9.9 | -21.9 | 1.7 | 173 | Significant stenosis at left middle cerebral artery |
| Patient 3 | 59 | female | 22.2 | No | No | Current | 68.2 | 6.2 | -18.4 | 2.55 | 270 | focal cerebral infarction at right temporal and parietal lobe |

BMI, Body mass index; CFR, coronary flow velocity reserve; E, early diastolic mitral inflow velocity; e’, early diastolic mitral annulus velocity; GLS, Global longitudinal strain; LVEF, left ventricular ejection fraction

**(2) Supplementary Figure and Figure Legend**

**Supplementary Figure 1. Correlation between CFR by MVd and exercise capacity**


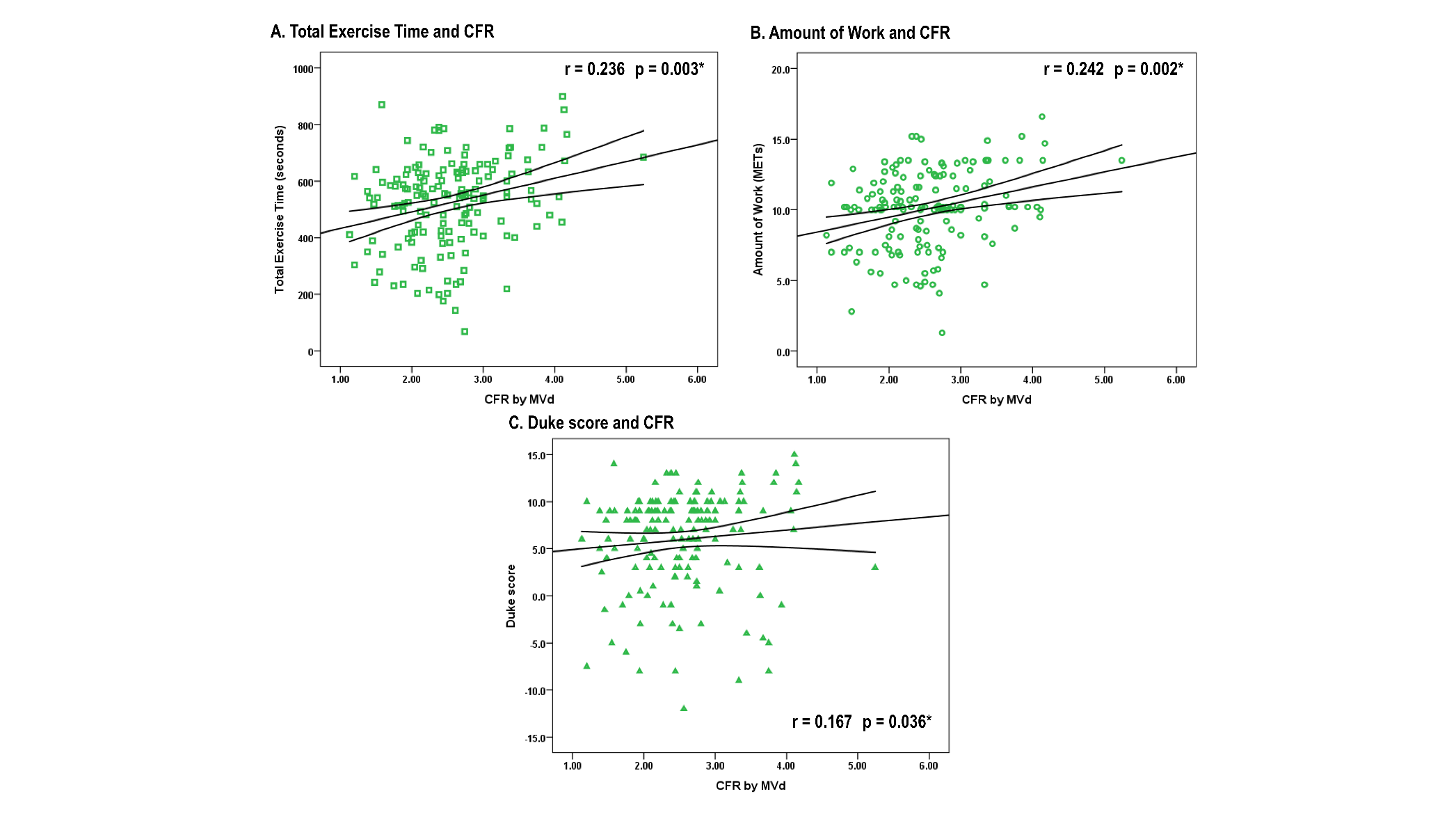
Abbreviations: CFR, coronary flow reserve; METs, metabolic equivalent of task; MVd, mean diastolic velocity of coronary flow.

**Supplementary Figure 2. Correlation between CFR by MVd and exercise capacity according to sex**


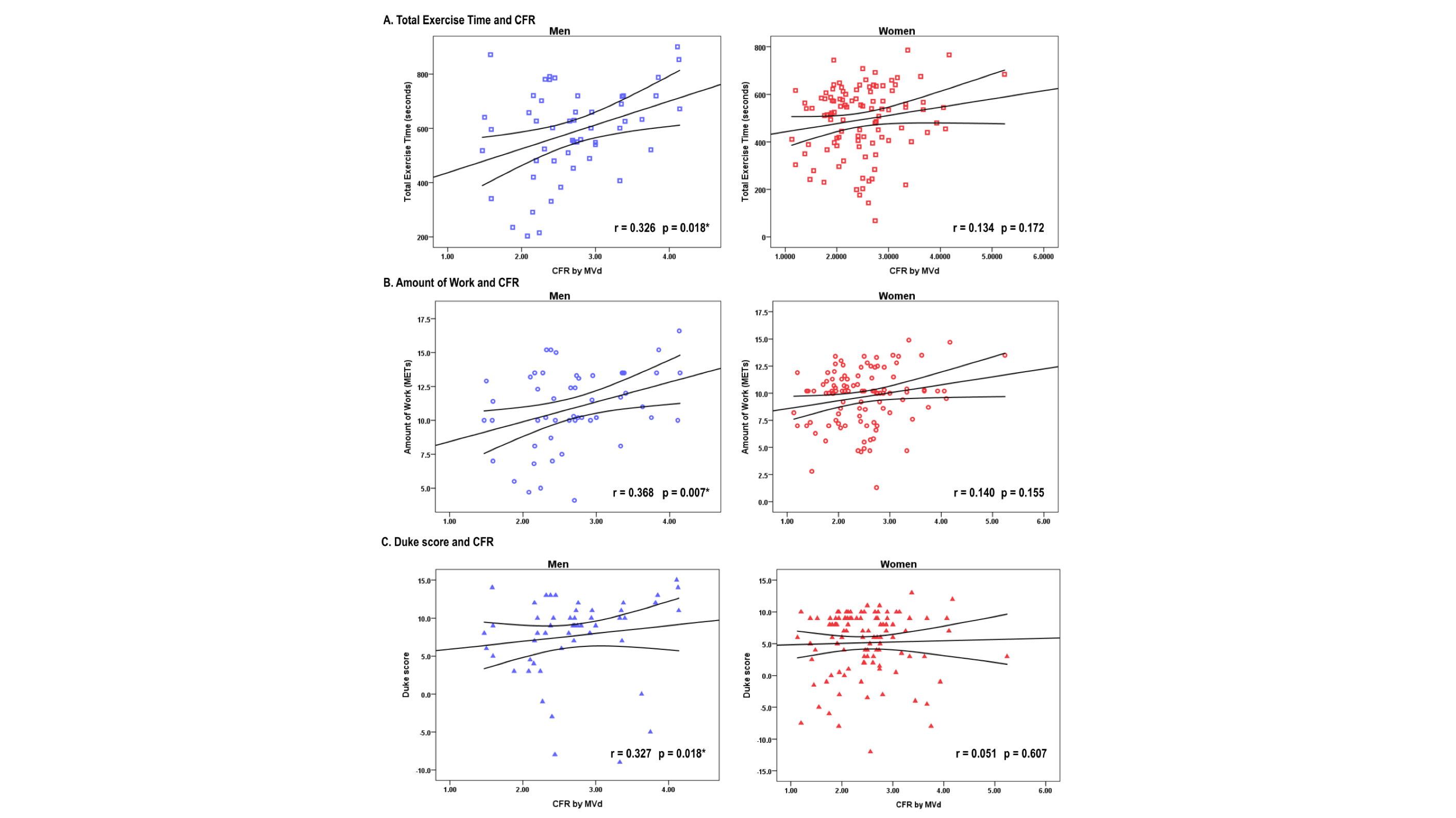


Abbreviations: CFR, coronary flow reserve; METs, metabolic equivalent of task; MVd, mean diastolic velocity of coronary flow.
